# Acceptability and Feasibility of a Nurse-Led, Community Health Worker Partnered Latent Tuberculosis Medication Adherence Model for Homeless Adults

**DOI:** 10.3390/ijerph17228342

**Published:** 2020-11-11

**Authors:** Benissa E. Salem, Erin Klansek, Donald E. Morisky, Sanghyuk S. Shin, Kartik Yadav, Alicia H. Chang, Adeline M. Nyamathi

**Affiliations:** 1 School of Nursing, University of California, Los Angeles, CA 90095, USA; dmorisky@ucla.edu; 2 Medical Education, University of California, Irvine, CA 92697, USA; eklansek@uci.edu (E.K.); ssschin2@hs.uci.edu (S.S.S.); kartiky@hs.uci.edu (K.Y.); anyamath@hs.uci.edu (A.M.N.); 3 Los Angeles County Department of Public Health, Tuberculosis Control Program, Los Angeles, CA 90007, USA; alchang@ph.lacounty.gov

**Keywords:** 3HP, latent tuberculosis infection (LTBI), nurse-led, community health worker (CHW)

## Abstract

Homeless adults are at increased risk of latent tuberculosis infection (LTBI), which can lead to active tuberculosis (TB) disease. The purpose of this study was to assess acceptability and feasibility of a six-month, nurse-led, community health worker-partnered short-course treatment (3HP) LTBI adherence model for a high risk, LTBI positive, homeless population. Informed by our community advisory board (CAB) and community-based participatory research principles (CBPR), a qualitative study was undertaken and used focus group discussions to identify perspectives of homeless men and women who had undergone LTBI treatment (N = 11, M_age_ = 51.2, SD 8.60, range 35–60). Three themes formed, which were engaging and recruiting LTBI intervention participants, delivering an LTBI intervention, and retaining LTBI intervention participants. Within those themes, barriers (e.g., lack of LTBI treatment readiness, substance use, etc.), and facilitators (e.g., LTBI and TB health education, familiarity with homeless population, etc.) were discussed to facilitate program recruitment, program delivery and program retention. These findings provide a greater understanding of how to effectively utilize a nurse-led, Community Health Worker (CHW) intervention delivery method to not only improve 3HP LTBI medication adherence, but also decrease substance use, improve mental health, and decrease unstable housing among this vulnerable population at high risk for active tuberculosis.

## 1. Introduction

Tuberculosis (TB) is caused by *Mycobacterium tuberculosis* bacteria [1] and is associated with poverty and homelessness [2]. In the United States (US), as compared to the general population, homeless persons have a 10-fold increase in TB incidence [3]. In Los Angeles County, approximately 7.6% of TB cases occur among those experiencing homelessness [4]. Prior to a TB diagnosis, over 5% of individuals report being homeless within the last year [5]. As compared to the general population, homeless populations are at higher risk for recent TB transmission [6,7,8] due to lack of TB treatment completion, malnutrition, and substance abuse (e.g., alcohol use) [5,9]. Additional risk factors include residing in a homeless shelter with persons with active TB disease [7] and length of stay in shelters [10].

As part of the US strategy for TB elimination [11], a key component is identifying and treating latent tuberculosis infection (LTBI) because five to ten percent of individuals with LTBI progress to active TB disease [1]. The United States Preventive Services Task Force (USPSTF) provides a grade B recommendation for LTBI screening with particular attention among those with additional TB risk factors [8] such as homelessness.

## 2. Latent Tuberculosis Infection (LTBI) Diagnosis, Risk Factors and Treatment Regimens

Latent Tuberculosis Infection (LTBI) is diagnosed when there is a positive skin or blood test for *M. tuberculosis*, a normal chest radiograph and no symptoms or physical findings suggestive of TB [12]. One of the approaches taken with LTBI testing is to focus on those who are at risk for progression from LTBI to TB disease [13]. Although LTBI positive individuals have a 5% to 10% risk of progressing to active TB disease during their lifetime [14,15], the risk is significantly higher in the homeless population. This risk can be mitigated by completing LTBI treatment; however, lack of LTBI treatment adherence remains consistently low in the US [16] and one of the biggest obstacles to TB elimination.

There are four treatment regimens for LTBI of varying duration (i.e., 3–9 months), including Isoniazid (INH) for 6 or 9 months, Rifampin for 4 months, and Isoniazid-Rifapentine (3HP) for 12 weeks [17]. Compared to the 9-month Isoniazid treatment, 3HP has a higher treatment completion rate and is as effective in TB prevention [18,19]. Among homeless populations, little is known regarding treatment adherence for shorter LTBI treatment.

## 3. Impact of 3HP LTBI Medication on TB Prevention among Homeless Adults and Other Vulnerable Populations

While a major challenge in TB prevention among persons experiencing homelessness is LTBI treatment acceptance and treatment completion [20], new models of LTBI intervention have been utilized with success. In one recent study of persons experiencing homelessness (N = 393, Median_age_ = 50, age range 13–74), 76.6% completed the 3HP LTBI treatment regimen [21]. Challenges with treatment adherence include following-up with persons experiencing homelessness because of relocation, mistrust, and alcohol or drug use [21]. Another successful model using nurse-led case management and incentives (NCMI) with education and tracking has proven effective in LTBI treatment among homeless populations in Central City East (Skid Row) in Los Angeles, California [22,23,24]. In a prospective, two-group, site randomized study which compared two groups (i.e., NCMI versus Control) among homeless adults (N = 520) with LTBI, there was a higher LTBI completion rate among the NCMI versus the control group with standard care and incentives (62% versus 39%) group [24]. These findings demonstrate potential in the utilization of NCMI among this vulnerable population.

## 4. Purpose

The purpose of this study was to identify perspectives of homeless adults, who had undergone LTBI treatment, integrate those perspectives in the development of a multicomponent intervention to increase 3HP acceptance and treatment completion, and assess acceptability and feasibility of the intervention.

## 5. Methods

### 5.1. Design

Informed by our community advisory board (CAB) and community-based participatory research principles (CBPR), a qualitative study was undertaken to engage homeless men and women with self-reported LTBI in two focus groups. Using a semi-structured interview guide (SSIG) the focus groups sought to identify the participants’ perspectives on barriers and facilitators of 3HP medication adherence. The SSIG focused on access to health services, experiences with LTBI treatment completion, and assistance in the design of the study. Some questions included, “Please tell us what health services you have sought in the last few years?” and “What would you say are the biggest barriers to completing the 12-week therapy that would prevent getting sick with TB in the future?” Another question included, “How can the community health worker (CHW) and nurse be most supportive to you in successfully completing the treatment in the community?”

### 5.2. Sample and Site

A total of eleven homeless men and women were enrolled from two community-partnered sites based on the following eligibility criteria: (a) aged 18–60 years of age, (b) received LTBI treatment over the last year (i.e., completers and non-completers), (c) experienced homelessness for at least six months and (d) experienced at least one mental illness, substance use, received medical care while homeless, and (e) able to speak English.

### 5.3. Brief Description of 3HP LTBI Nurse and CHW Intervention Program

The 3HP LTBI Nurse and CHW six-month intervention is composed of a community-based, research nurse (RN) and CHW. After the first dose of 3HP, the CHW conducts a weekly 20-min case management session over 12 weeks. Every week, the CHW will deliver all components of the program as well as assess 3HP side effects, track missed doses, and provide health-related support. The clinic-based primary care provider is involved in decision-making for ongoing treatment.

In week one, the 20-min case management session focuses on detailed information about the program, support to identify their personal values, delineate personal participant goals, education about TB and barriers to adherence, including drug and alcohol use. In weeks two–three, the 20-min case management session focuses on the dangers of substance use, support in dealing with mental health problems, and facilitation of referrals for outpatient substance use, mental health counseling, stable housing, and job skills, etc. In weeks four to twelve, CHWs focus the 20-min case management session on facilitating the completion of 3HP, reducing the urge to use drugs and alcohol, seeking stable housing and health care access, and continuing to identify triggers for non-adherence of 3HP.

## 6. Procedures

Institutional Review Board (IRB)-approved recruitment flyers were posted in community-based partner organizations. In a screened and quiet location, participants who were interested in learning more information regarding the study participated in information sessions with the research team who went over the basics of the study and answered questions. Oral screening consent was obtained prior to the screening. Thereafter, a study information sheet (written summary—waiver approved by the IRB) in lieu of signed informed consent was administered to each eligible participant and sufficient time was provided to consider whether to participate in the research. After allowing the potential subject time to read the study information sheet, any additional questions were answered by the research team and an oral verbal agreement to participate in the research was obtained; the participant was enrolled in a 45-min focus group discussion with 4–5 other participants. Participants were compensated $3 for completing the screening and $15 for the focus group discussion (if eligible).

## 7. Data Analysis

The trustworthiness of data was ensured by applying principles of credibility, transferability, dependability and confirmability to data collection, analysis, interpretation, and dissemination [25]. In this study, to ensure credibility (e.g., how closely findings match reality) [25], the researchers had early interactions with the community-based research site, participants, and their environment. In addition, frequent debriefing sessions between the research team allowed for peer scrutiny of the research project. Additionally, audio recordings were used to assess the validity of the focus group transcripts. The transcriptions were then coded line-by-line and analyzed by the first author and research assistant. Through coding, major themes were discovered, highlighted, and discussed between them.

In this study, transferability (e.g., generalizability) [25] was ensured by disclosing the types of organizations involved, inclusion criteria, number of participants, how data was collected, frequency and time of data collection which will allow for comparison and generalizability. For dependability (e.g., reliability) [25], the researchers specified how data was collected (i.e., focus groups) and the geographic location (i.e., Central City East) was specified [25]. Finally, confirmability (e.g., objectivity so that the participants ideas are reflected) [25], was ensured by developing an audit trail which included documenting meetings, developing a study timeline and weekly progress report, etc.

## 8. Results

### Sociodemographic Characteristics

Table 1 reports sociodemographic characteristics of the sample (N = 11, M_age_ = 51.2, SD 8.60, range 35–60). The majority were men (90.0%) and self-reported being Black/African American (45.5%), followed by White (45.5%) and other (9.1%); over one-third (36.4%) identified with being Hispanic and/or Latino. The majority of the participants (54.5%) were born in the United States, followed by Mexico (27.3%) or other (18.2%). Over half (63.6%) had children. Over one third identified as Catholic (36.4%), other (36.4%) or Protestant (27.3%) as their religious affiliation. The majority of participants had completed less than 12 years of education (36.4%), with 27.3% completing > 12 years of education, 27.3% college/post high school (27.3%), and 9.1% completing graduate school.

**Figure 1:**
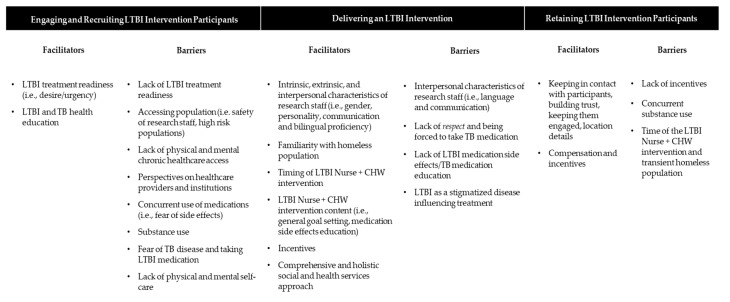
Qualitative themes and subthemes of nurse led, CHW LTBI adherence model for homeless adults (N = 11).

Table 2 reports acceptability and feasibility of the 3HP LTBI program in 3HP Delivery among homeless adults diagnosed with LTBI. Overall, the majority of participants (90.9%) felt that the program addressed experienced challenges to complete LTBI medication treatment programs, followed by 9.1% who felt that the program somewhat addressed the issue. The majority (63.6%) of participants felt that the session identified substance use challenges and/or discussions of substance use and its challenges. Less than half (45.5%) of participants self-reported that the proposed intervention program identified challenges faced by someone experiencing mental health issues. Nearly three quarters (72.7%) of participants felt that the discussion included useful information on stable housing, while 27.3% felt it was only somewhat included. All the participants (100%) felt that the program would be relevant to both men and women, that the discussion helped improve the program, and that they would recommend the program to those with LTBI. Figure 1 pictorially depicts the major themes and subthemes which include barriers and facilitators to LTBI Nurse and CHW Program recruitment, delivery, and retention.

**Theme 1:** Engaging and recruiting LTBI intervention participants

Homeless adults shared **facilitators** and **barriers** to engaging and recruiting the community. Facilitators to engagement and recruitment included LTBI treatment readiness (i.e., desire/urgency) and LTBI and TB education. Across the focus groups, homeless participants shared that it was important for the research team to “motivate people” and to “...catch them at the right time [when they are] ready to surrender.” As one individual noted,

...If they want to stay out of harm’s way, it’s best to get and finish [LTBI treatment] where you can help yourself and help others around you. Because once you’re exposed, there’s nothing else to do… See, and myself, I did what I had to do...

*Participant 4, Focus Group 2*

While another participant shared that “fear works” and there was urgency related to LTBI treatment, another participant reflected on fear associated with having to leave a shelter due to a TB diagnosis. This participant reflected,

When they give me [LTBI medication], I was like all scared and all panicked. Oh, my God, hopefully I don’t got TB because the only thing you think about is …you got to leave this place. You can’t be in this shelter.

*Participant 9, Focus Group 3*

Across the focus groups, homeless participants shared that LTBI and TB health education was important so that homeless adults could recognize that they could be impacted by TB and LTBI and that more information about these conditions was needed. In particular, one participant described that television health education messaging would be successful.

...There’s a commercial about stopping smoking. But, the lady is smoking cigarettes and then you see her later on with a hole in her throat… And, that’s the way you have to come at people the same way you just--, you got to let them know, this could be you. Already have them in a casket. That still works.

*Participant 5, Focus Group 2*

He also described the lack of knowledge related to test results,

…down the line, you can actually die from [TB] which I already knew … Because like I say, I’ve been showing up [PPD] positive since I was a little kid. I never thought about, you know, when they always say, okay, you’re negative after a chest x-ray. I think that was just something I was going to have to do every time I take a TB test.

*Participant 5, Focus Group 2*

Homeless participants discussed **barriers** to engaging and recruiting the community which included lack of LTBI treatment readiness, accessing the population (i.e., safety of research staff, high risk populations), lack of physical and mental chronic healthcare access, perspectives on healthcare providers and institutions, concurrent use of medications (i.e., fear of side effects), substance use, fear of TB disease and taking LTBI medication and lack of mental and physical self-care.

Lack of LTBI treatment readiness impacts engaging and recruiting the community. One participant noted, “…you can lead a horse to water, but you can’t make them drink.” As another individual reflected,

I mean it’s a great thing you guys doing this but, most of the people don’t want to really get help around here.

*Participant 9, Focus Group 3*

Homeless participants shared that when accessing and engaging the homeless population in the community, it is important to be safe, especially if some participants are “in a life of prostitution, in the life of gang banging...” Some of the **barriers** to engaging and recruiting the community include the lack of paperwork related to identification (i.e., *identification, social security*, etc) and health insurance. One individual noted,

...There [are] a lot of people that are afraid to go to the doctor because they’re afraid that they’re going to have to pay something.

*Participant 10, Focus Group 3*

Additional **barriers** to engaging and recruiting the community which were described by homeless participants included perspectives on healthcare providers and institutions which embodied the lack of institutional trust; further, they shared their perspectives on healthcare providers and institutions. One participant noted,

…People think, you know, I don’t like doctors. I ain’t going to lie. I don’t like going to the hospital or nothing. So, people have that in their head. Oh, the doctor? No, no. Because I’ve been told, I got to go to the doctor. I’m the same way but since I’m not on, you know, crazy people on drugs.

*Participant 1, Focus Group 2*

Across the focus groups, homeless participants shared that taking concurrent medications could be difficult because of a fear of side effects to the LTBI medication and other current medication they are taking.

That’s what stops me from doing it if you talk about medicine because I be thinking about the side effects of it, you know. You know, but let them know that there’s nothing going to hurt you.

*Participant 5, Focus Group 2*

Across the focus groups, homeless participants shared that there is TB-related fear or fear in general which can impact behavior; in particular, one participant said, “People with fear is, you know, be like a stone.” Others shared that when using substances, “drugs make you forget about anything you on. You don’t care nothing about getting well.” Homeless participants also shared that there is a lack of physical and mental health self-care. One individual reflected,

For whatever reason, they just, I don’t want to say they don’t care. I’m thinking a lot lost how to care.

*Participant 8, Focus Group 3*

**Theme 2:** Delivering an LTBI intervention

Homeless individuals shared **facilitators** and **barriers** to delivering an LTBI intervention. Facilitators included intrinsic, extrinsic and interpersonal characteristics of research staff (i.e., gender, personality, communication and bilingual proficiency), familiarity with homeless population, timing of LTBI Nurse and CHW intervention, LTBI Nurse and CHW intervention content (i.e., general goal setting, medication side effects education), incentives, comprehensive and holistic social and health services approach.

Across the focus groups, homeless participants shared those intrinsic, extrinsic, and interpersonal characteristics of research staff that would impact completion of the intervention program. Gender was discussed, with some disagreements on if it was important or not to program delivery. On the one hand, some participants shared that gender did not matter if they were seeking help, with one male participant sharing,

I think that if ... I have like depression, I suffer from depression or anxiety, you know, the thought process is I need help, I need help… It doesn’t matter who’s going to help me.

*Participant 5, Focus Group 2*

On the other hand, a participant shared his experience working with women in this population and stated,

…I’ve talked to some battered women and worked in a facility where they [were] housed and yeah, trust, yeah, it’s harder. But...if it’s coming from a place of letting them know, look, we’re here to help you.

*Participant 1, Focus Group 2*

Participants also shared that personality was an important facilitator to delivering an LTBI intervention. Participants mentioned that research team must “[earn] that trust” and that they “have to press the issue” to help participants complete treatment.

Additional facilitators which were discussed included the importance of communication and building rapport with the research team. Participants emphasized the importance of communication with one participant stating,

So, they can have like a thing in common so people can really get cured. They can get the thing that they’re there for, you know, take the treatment.

*Participant 9, Focus Group 3*

Across the focus groups, homeless participants shared that familiarity with homeless population was a facilitator to completion of the Nurse and CHW intervention. Participants stated the importance of having a research team who “know[s] the streets.” Another participant shared,

...That’s the risk you take when you’re trying to help people. So, like I say, you just got to get out there, you know and make an effort … and then have people like myself and whoever, not shy, they ain’t scared to talk about it. You get us out there and we relate to them.

*Participant 1, Focus Group 2*

Another participant shared how having people who are familiar with the homeless population can help the delivery of the intervention by stating,

That’s the best way and like people like ourselves, me...have us go with you, you know. I used to use drugs or used to... Put us on the screen or TV. Not saying that that’s where I want to be but I’m not ashamed of nothing about myself.

*Participant 5, Focus Group 2*

Across the focus groups, homeless participants also shared that the timing of the LTBI Nurse and CHW intervention would be a facilitator to the intervention. Participants shared days that participants would be less likely to engage, including the weekends, or directly after receiving their benefits, which is the first 10 days of the month. In particular, participants shared that Tuesdays, Wednesdays or Thursdays would be best. One participant stated,

You don’t want to do it Fridays because Friday it’s like end of the week. Most people, in my opinion, like, you know what I’m saying, like Fridays see like oh, the weekends coming or by like the middle of the week, you know, Wednesdays.

*Participant 9, Focus Group 3*

Across the focus groups, homeless participants discussed facilitators of LTBI Nurse and CHW intervention content (i.e., general goal setting, medication side effects education). Homeless participants shared how having a friendly provider helps build trust. One participant reflected about the importance of being involved in his care. He shared,

A goal, a homework assignment or something that I can look forward to. If I’m going to get better, if I want to get better... this is what I can do to help. Something to look for, give me a goal. Give me something.... What is TB? What is tuberculosis? Give me something I can study up on and learn about. I can’t just talk about it...

*Participant 1, Focus Group 2*

Participants noted that medication side effects education was critical; in fact, one participant shared the importance of knowing about side effects and that the 3HP LTBI medication is “not going to hurt you.” One participant reflected,

I think the side–that was my first question. Because, you know, side effects, I see people take–they advertise medicine on TV like you guys are. And they come up with this long list of side effects. It sounds like I’m better off not taking it. So, that’s the key, you got to let them know and I take it. I take your medicine. Right now, I’m still on and I’m doing it for another month and a half. But that’s–I take a whole handful of pills at one time...

*Participant 2, Focus Group 2*

Another participant shared concerns about combining medications and shared,

...I’m already taking medication already for other illnesses, you know what I’m saying, what side effects is this going to–it could coincide with me. If I’m still drinking and using or I’m taking, or if I’m on it, I have HIV. I’m HIV positive. And I’m taking this other medication and I’m doing drugs, too, come on. That’s a battle in itself. That’s a battle.

*Participant 4, Focus Group 2*

Participants shared how a lack of LTBI medication education side effects/TB medication education could be a barrier to treatment. Homeless participants noted that a lack of education regarding the medication’s benefits was a barrier to continuing treatment, with one participant stating,

… He go to the clinic and they tell you to take this, take that but they don’t really explain it to you, what would be the benefits and all that.

*Participant 9, Focus Group 3*

Other participants stated that there is a lack of education surrounding the administration of TB medication and compliance. He shared about his fiancé, stating,

She didn’t want to comply. So they took her to put her in the hospital where she could take her medicine… See, a lot of people are not aware of that.

*Participant 5, Focus Group 2*

Other participants shared the importance of providing incentives as well as a comprehensive and holistic social and health services approach. In particular, participants shared how including mental health services, housing services, chronic disease (i.e., Diabetes), vision (i.e., eyeglasses) would be beneficial to the program’s participants. One person shared,

Can we work on housing? Can we work on that? Can we work on the depression?”

*Participant 1, Focus Group 2*

Another participant shared why it was important to treat more than just TB infection by explaining,

Yeah, very, very important and in Skid Row too much people problems mental.

*Participant 12, Focus Group 3*

Homeless participants across the focus groups also discussed how interpersonal characteristics of research staff (i.e., language and communication) could be a **barrier** to delivering an LTBI intervention and 3HP LTBI treatment completion. One participant noted the connection between language and building trust,

That’s a big barrier. Like people don’t really speak English and that’s a big cultural issue. If you don’t speak Spanish to them, they’re not really going to trust you.

*Participant 9, Focus Group 3*

Additional barriers include how a lack of respect and being forced to take TB medication could be a **barrier** to delivering an LTBI intervention and 3HP LTBI treatment completion. Participants shared that, “being forced” and having no choice inhibits their desire to take the medication as prescribed to them. One participant stated,

…Once you cross a certain barrier line, once you hear a person telling you you’re forced to take it, forced to do something, forced to take the medication, you cross that invisible line.

*Participant 2, Focus Group 2*

Homeless participants also shared concerns that people with active TB did not know that being forced to take the medication could be a consequence of non-compliance with one participant noting,

That’s what happened with my fiancé I was with. She didn’t know and she didn’t want to take nothing. So, they had to incarcerate her to get her to take it. And she start taking it. After while they let her out but she had to comply. She didn’t want to comply. So, they took her put her in the hospital where she could take her medicine. And she didn’t like. She didn’t comply. See, a lot of people are not aware of that.

*Participant 5, Focus Group 2*

Homeless participants also discussed how disease is stigmatized and this can influence how they accept treatment; in particular, the nature of TB being a stigmatized disease affects treatment of LTBI and TB as it will not be taken openly. One participant explained that taking medication was not desired and there was difficulty sharing the disease status with others. One participant shared,

When you’re in that element or you’re in that lifestyle, a lot of people, you tell a person they have a disease, a disease, they’re not going to respond to, really, they’re not going to–they’re going to, I mean, they’re going to treat it but it’s going to be a hush-hush thing. It’s going to be a, I’m not going to tell my friends. I don’t want nobody really to know.

*Participant 3, Focus Group 2*

Homeless participants also shared that many participants may have difficulty with taking pictures due to lack of trust and fleeing certain environments. In fact, some avoid major holiday events in Skid Row to avoid the multitude of individuals taking pictures. One participant noted,

I think that’s going to be a very hard issue for like the young adults. Like, people over, like a little bit over 20 because some of them are teenage runaways and they stay down here. And they don’t want their picture being posted anywhere because that’s going to be a major issue for them.

*Participant 4, Focus Group 2*

**Theme 3:** Retaining LTBI intervention participants

Homeless participants shared **facilitators** and **barriers** to retaining LTBI intervention participants. Facilitators included keeping in contact with participants, building trust, keeping them engaged, location details, compensation, and incentives. Across the focus groups, homeless participants shared that keeping in contact with participants beyond the immediate delivery of the program was important. One participant shared,

… It’s not just a hi, bye, thing, you know what I’m saying. And make sure they continue getting to know, keep random coming down here, check in on them. Get their phone number.

*Participant 1, Focus Group 2*

Participants also shared that building trust between provider and participant was necessary to encourage retaining LTBI intervention participants. One participant stated,

Like me, it doesn’t really matter. But, like take the medication, I don’t know if I’m right or wrong. It’s a cultural thing, too. Like, you know what I’m saying? Like, trust, when I went out there, there was this nurse.

*Participant 9, Focus Group 3*

Homeless participants shared how keeping participants engaged is also a vital step in retaining LTBI intervention participants and one that can be difficult to maintain. One participant shared the importance of keeping people engaged through the process, and the need to be aware that some people may not want to provide information. He said,

Keep them engaged. You know I’m saying? And a lot of people, and then people, your information, and people’s information, they’re really tired of giving–they don’t want to give up that information, you know. And they think that, and then you have to really, like I said, just baby steps before you.

*Participant 4, Focus Group 2*

Across the focus groups, homeless participants also discussed how compensation and incentives were facilitators to retaining LTBI intervention participation. Participants discussed how incentives are “a big motivation” and “an inspiration.” One participant reflected,

You giving me a whole handful of pills, yours might be less but I was like, you know, since I’m new, just getting here, I ain’t got no money. I was like oh, you get a card? Oh, really? Give it here. I’ll take the chance. That makes a big difference if it’s any kind of, like I said, what I’ve done and I still take it every Tuesday.

*Participant 2, Focus Group 2*

Barriers included lack of incentives, concurrent substance use, time of the LTBI Nurse and CHW intervention and transient homeless population. Across the focus groups, participants also discussed how a lack of incentives was a barrier to retaining LTBI intervention participants. In fact, participants shared that ongoing incentives was necessary, with one participant stating,

You quit giving, they’re going to stop coming.

*Participant 8, Focus Group 3*

Homeless participants also discussed how concurrent substance use was a **barrier** to LTBI Nurse and CHW program retention. Across the focus groups, homeless participants shared how concurrent substance use impaired retaining LTBI intervention participants. One participant stated,

...If they drinking or doing whatever they want to do, they ain’t trying to–they ain’t worrying about doing whatever. Like, taking medication. You got to hit them a day or a time that they’re willing to do that because you see them drinking or whatever they’re doing, even though they got–they got an appointment with you guys or they give you their word, they’re not going to honor that because they’re doing.

*Participant 9, Focus Group 3*

Homeless participants discussed how the time of the LTBI Nurse and CHW intervention and transient homeless population is a barrier to retaining LTBI intervention participants. Participants discussed how the timing of the intervention contributed to a lack of program retention. One participant noted,

People here, they going get their money in part or for like a week, one or two weeks and then come back. They stay in the area but you won’t see them because a lot of people go to hotels and do things like that.

*Participant 9, Focus Group 3*

Lastly, participants shared that the transience of the population is a barrier that is difficult to overcome. One participant shared,

Yeah, you got to show urgency. You need to–it’s hard to keep them in one place.

*Participant 8, Focus Group 3*

## 9. Discussion

Homeless participant’s shared specific **facilitators** and **barriers** related to engaging and recruiting participants into a LTBI intervention, delivering an LTBI intervention, and retaining LTBI intervention participants. Facilitators for engaging and recruiting participants into a LTBI intervention are divided into desire to change and LTBI and TB disease education. Being highly motivated to complete LTBI treatment has been found to increase treatment completion [24]. As TB is a stigmatized disease, many participants may confuse active TB with LTBI which is exacerbated by fear of being asked to leave the shelter system or being forced to take medication for active TB. For the research team, it is important to utilize creative teaching strategies (e.g., health education games,) which can be tailored for low health literacy clientele to understand TB and LTBI differences and encourage participation.

Additional barriers were noted related to engaging and recruiting participants; in particular, during the recruitment process, it is critical to train research staff members to work with others who have in-depth knowledge and firsthand experience with the community. Participants also described that a barrier to engaging and recruiting participants include those who are taking concurrent medications due to fear of side effects. Thus, partnering with a community-based clinic to refer participants to clinicians can clarify clinical questions and dispel myths. Relatedly, participants felt that substance use was a competing priority; thus, the research team needs to refer participants to resources using evidence-based substance use resources (e.g., screening, brief intervention, and referral to treatment) [26].

Homeless participants discussed facilitators to successful LTBI intervention completion, which included research staff qualities (e.g., men and women with lived experiences and familiarity of homelessness, good role modeling) and bilingual proficiency (e.g., Spanish and English). Relatedly, homeless participants also discussed the importance of timing of the intervention to optimize screening to enrollment success. For instance, in this community, Tuesdays–Thursdays are more successful for screening to enrollment compared to Mondays, the first 10 days of the month, or the weekends.

Homeless participants also discussed the importance of intervention content recommending that it should integrate personal goal setting, education related to active versus LTBI, education on medication side effects, and a more comprehensive holistic social and health services approach (e.g., physical and mental health), as well as housing resources. Previous research has explored the use of TB education sessions concurrently with TB treatment [24]. Introducing goal-oriented tasks to support patient autonomy in the treatment process may influence treatment outcomes. Relatedly, participants shared how the disease may be a “hush-hush” issue; thus, participants may not want to disclose participation or results to others (i.e., family, friends, community, etc.).

Homeless participants discussed facilitators related to retaining LTBI intervention participants including keeping in touch with participants and keeping them engaged, building trust, and providing incentives (e.g., gift cards, etc). A patient-centered approach and a positive provider and client relationship improves TB treatment completion [27] and needs to be rooted in maintaining contact and building trust with the research team. A locator guide is one tool which can be employed to obtain location details (e.g., hang outs) and follow-up with clientele. Using this method, our previous research tracked participants over 6 months, which supported a high retention rate (87.7% to 90.1%) [28]. Our previous research further utilized non-coercive incentives throughout the research process to aid in study completion [24,28,29].

Future LTBI programs focusing on homeless populations need to include compensation and incentives, along with tracking tools. Additional barriers to retaining LTBI intervention participants included the concurrent use of alcohol and drugs and the transient nature of the population. The monthly federal benefit distribution challenges retaining and finding participants in the first 10 days of the month. Thus, it is important to consider the timing of all program activities in future LTBI treatment programs.

While there are a number of notable strengths in this study, some limitations include the number of participants in the focus groups, disproportionate representation of men from a single site, and language barriers which hindered communication between the research team and participants. Altogether, despite these limitations, this present study reflects the perspectives of a heterogeneous population that provided insight into cultural and behavioral decision-making, and shed light on central issues on how to effectively utilize a Nurse–CHW partnered intervention delivery method to improve 3HP LTBI recruitment, delivery, retention and completion among this vulnerable population at high risk for active tuberculosis.

## Figures and Tables

**Table 1:** Sample characteristics of homeless adults (N = 11).

	Mean	SD, Range
Age	51.18	8.60, 35–60
Number of Children	2.43	2.23, 1–7
	**N**	**%**
**Gender**		
Male	10	90.9%
Female	1	9.1%
**Race**		
Black/African American	5	45.5%
White	5	45.5%
Other	1	9.1%
**Ethnicity**		
Not Hispanic or Latino	7	63.6%
Hispanic or Latino	4	36.4%
**Country of Birth**		
United States	6	54.5%
Mexico	3	27.3%
Other	2	18.2%
**Children**		
Yes	7	63.6%
No	4	36.4%
	**N**	**%**
**Religion**		
Catholic	4	36.4%
Other	4	36.4%
Protestant	3	27.3%
**Educational Status**		
<12 years	4	36.4%
>12 years	3	27.3%
College/Post High School	3	27.3%
Graduate School	1	9.1%
**Homeless Status, Ever**		
Yes	11	100%
No	0	0%

**Table 2:** Acceptability and feasibility of a nurse led, CHW LTBI adherence model for homeless adults (N = 11).

	A Great Deal		Somewhat		Not At All	
	N	%	N	%	N	%
Did the session bring up the challenges homeless adults face when attempting to complete a medication treatment program?	10	90.9%	1	9.1%	0	0%
Did the session identify the challenges experienced by someone with a substance use problem?	7	63.6%	3	27.3%	1	9.1%
Did the session identify the challenges experienced by someone who is experiencing a mental health issue?	5	45.5%	5	45.5%	1	9.1%
Did the session bring up useful information on strategies that can help you if you needed more stable housing?	8	72.7%	3	27.3%	0	0%
Do you feel this program would be relevant for men as well as women?	11	100%	0	0%	0	0%
Do you feel the session was conducted in a useful and understandable way?	11	100%	0	0%	0	0%
Do you feel the discussion helped to improve the program?	11	100%	0	0%	0	0%
Overall, would you recommend this program to other persons with latent TB who need to complete a medication treatment program.	11	100%	0	0%	0	0%
